# *Lindernia dubia* (L.) Pennel as an Alien Weed in Central Spain: A Case Study

**DOI:** 10.3390/plants13131859

**Published:** 2024-07-05

**Authors:** María Dolores Curt, Gema Sánchez, Pedro Luis Aguado, Inés Santín-Montanyá

**Affiliations:** 1 Department of Agrarian Production, Universidad Politécnica de Madrid (UPM), Calle Ramiro de Maeztu 7, 28040 Madrid, Spain; gema.sanchezc@upm.es (G.S.); pl.aguado@upm.es (P.L.A.); 2 Department of Environment and Agronomy, Instituto Nacional de Investigación y Tecnología Agraria y Alimentaria (INIA-CSIC), 28040 Madrid, Spain; isantin@inia.csic.es

**Keywords:** *Lindernia dubia*, invasive species, plant traits, cattail seedlings, constructed wetlands

## Abstract

*Lindernia dubia* (L.) Pennell is a species with invasive behavior outside of its native range of distribution (America), linked mainly to aquatic habitats. This annual species has been acknowledged as a weed in rice paddies in Europe and Asia. Due to the impacts of this invasive plant, some authors have even listed this species as a global invader. The present work focused on spontaneous plant species occurring in seedlings of *Typha domingensis* Pers. grown in central Spain for the establishment of constructed wetlands. Weed inventory revealed the presence of *L. dubia* as a dominant spontaneous species in this crop environment. A suite of mesocosm experiments were designed to study the population density of *L. dubia* versus that of the other dominant plant species, and to determine traits associated with its weedy potential. The results showed that *L. dubia* presents competitive attributes such as morphological variability, early flowering, long seeding time, short growth cycle, small and light seeds and a high seed production and germination rate (25 °C), meaning a high reproductive capacity in a cycle of about three months for plant growth in non-limiting conditions. The data obtained from this work provide a basis for understanding the weedy potential of *L. dubia*, and for management decisions of a potentially invasive species, which has been little investigated in Europe

## 1. Introduction

*Lindernia dubia* (L.) Pennell (yellowseed false-pimpernel) is an annual herb of small size (stems 1.5–27 cm), native to America, where it grows in the borders of ponds, river banks and other moist-to-wet habitats [1]. Stems can be erect, ascending or prostrate, and they can root at the lower nodes; leaves are simple, opposite and sessile, and their shape is variable, from lanceolate to ovate or suborbicular [1]. Recently, the distribution of this species has expanded from America to various other regions of the World as distant as Europe, Korea or Taiwan [2,3]. Its flowers are gamopetalous, zygomorphic, chasmogamous or cleistogamous [4]; the fruit is a septicidal capsule with an ellipsoid or globose shape, containing numerous seeds which are ellipsoid or rectangular [1]. According to Lewis [1], this species shows a significant morphological plasticity, especially in terms of its vegetative characteristics. 

The Global Biodiversity Information database [3] mentions that there is evidence of the impact of this species in Italy, Romania and Japan. Specifically, in Europe, *L. dubia* (hereafter referred as LIDDU) has been identified as an invasive species in natural aquatic habitats of the Netherlands [5], Romania [6] and Italy [7].

In crop environments, LIDDU has been acknowledged as a weed in rice paddies, where its occurrence seems to be expanding [8,9,10]. Despite the fact that rice is a staple food crop, there is little information on the traits possessed by LIDDU that promote its spread and on its performance as a weed, probably because other weeds that affect rice, such as *Echinochloa* spp. and *Cyperus* spp., are much more harmful [11]. In the case of Europe, the cultivation of rice is concentrated in two countries, Italy (60.3%) and Spain (15.5%) (rice cropped in Russian Federation not included) [12], and in both countries LIDDU has been reported as an alien weed affecting rice [13]. In its native range—as a component of natural vegetation—LIDDU may play a certain role in the conservation of aquatic environments and the restoration of wetlands [14]. Additionally, LIDDU may also occur in other productive moist habitats, such as meadows, plant nurseries or treatment wetlands. 

Treatment wetlands are constructed aquatic habitats intended for the restoration of ecosystems or the management of wastewater; they are regarded as nature-based solutions in various contexts [15]. Among the most commonly used plants in constructed wetlands (CW) are *Typha* spp. [16]. *Typha* spp. can be established in constructed wetlands from rhizomes, seeds or seedlings; while the supply of rhizomes requires many resources (rhizome collection, logistics expenses), seeds are easy to collect and use. However, direct sowing is less successful than the establishment of seedlings, due to seed drift from water movement. This fact, along with the need for large number of plants (five to fifty individuals·m^−2^ depending on the type of wetland) [17], has placed the focus on nursery-propagated plants for CW establishment [18]. In this respect, proper sanitation is essential for producing marketable plants and preventing the spread of pests; some authors highlight that weed growth in container-grown nursery stock is a serious problem not only in terms of economic losses but also in terms of the risk of the spread of troublesome plant species, such as alien or invasive plant species [19]. In this regard, we hypothesized that helophyte nurseries may represent an environment prone to weed infestation, due to high availability of growth resources such as water and nutrients. To the best of our knowledge, the occurrence of weeds in helophyte nurseries has not been addressed in the published literature. 

In order to shed some light on spontaneous flora occurring in helophyte nurseries and the interspecific competition between dominant species, we set up a mesocosm experiment with the helophyte *Typha domingensis* Pers. (commonly known as cattail) intentionally grown from a seed in a nursery in Spain for constructed wetlands. The primary objectives were to develop an inventory of potential weeds in cattail nurseries and to assess the occurrence of the prevailing taxa and their introduction pathways in order to understand the source and species that should be controlled. In the course of species identification works, the alien species *Lindernia dubia* (L.) Pennel was recorded; this finding has encouraged research on the performance and characterization of this species. Thus, in this paper, (i) a case study of weeds in cattail seedlings is presented, (ii) a new record of the alien species *L. dubia* is reported, (iii) the distribution of dominant weeds is assessed and (iv) traits of *L. dubia*, a dominant alien species, are determined in order to build a dataset for the estimation of its weedy potential.

## 2. Results

### 2.1. Weed Inventory and Assessment of Dominant Species

**Figure 1:**
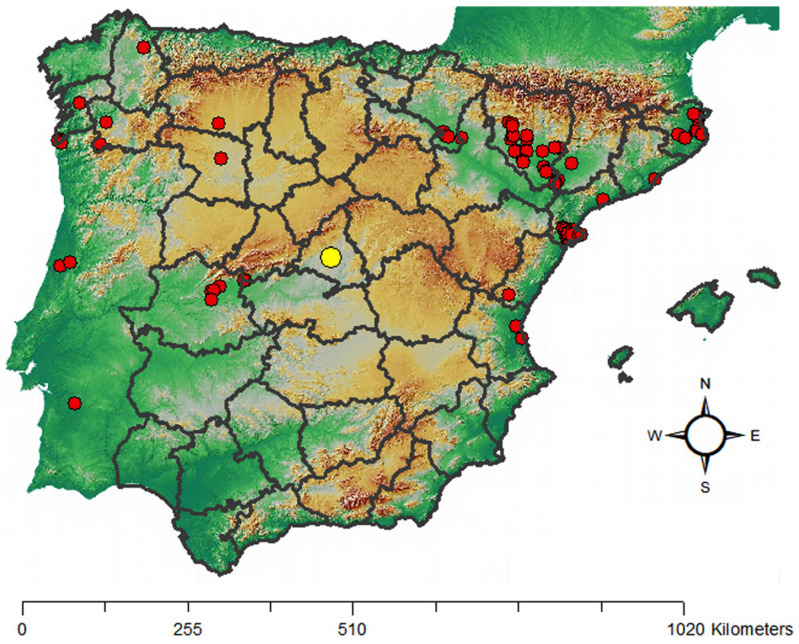
Confirmed records of *Linaria dubia* (L.) Pennel in the Iberian Peninsula (red dots), according to the Information System of the plants of Spain [21]. (Simplified map). Yellow dot = Madrid.

Plant species identified as weeds of cattail seedlings and their constancy values are shown in Table 1. In all, there were five families, Linderniaceae, Brassicaceae, Asteraceae, Cyperaceae, Onagraceae and Poaceae, represented in this particular environment, including, five annual species (in four families) and four perennials (in three families) per life cycle. The Poaceae family presented taxa in both categories; the annuals *Echinochloa crus-galli* (L.) P. Beauv. (ECHCG; common name: barnyardgrass) and *Digitaria sanguinalis* (L.) Scop. (DIGSA; hairy crabgrass) and the perennial *Agrostis stolonifera* L. (AGSST; creeping bentgrass). Per their native range, three alien species were recorded, including *Lindernia dubia* (L.) Pennel (LIDDU; yellowseed false pimpernel), *Cyperus eragrostis* Lam. (CYPER; tall flatsedge) and *Cyperus rotundus* L. (CYPRO; nutgrass), according to the list of alien species in Europe provided by EASIN [20] (CYPRO is considered partly native to Europe). The occurrence of LIDDU represented a new record of this alien species in Madrid (Figure 1) [21].

LIDDU was the most frequent species in the flooded seedling trays, showing a rate of constancy of 0.9828 (Table 1), while CARHI was second in the rank (0.9425 constancy). LIDDU and CARHI by far surpassed the occurrence of other species, which presented a <0.34 constancy. The existence of dominance was also confirmed by the estimate of the Simpson index (D = 0.73), while the estimate of the Shannon index (H = 1.53) indicated a low diversity.

**Figure 2:**
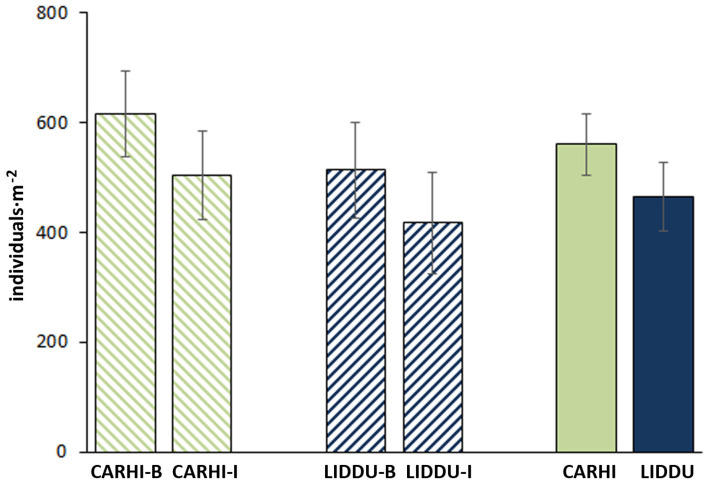
Population density of dominant plant species. CARHI =* Cardamine hirsuta* L.; LIDDU = *Lindernia dubia* (L.) Pennel; B = border position; I = inner position. The bars show ± standard error.

Population density by dominant species and tray position are shown in Figure 2. In total, the population density ranged from a minimum of 606 to a maximum of 1543 individuals·m^−2^, showing a mean value of 1024 ± 263 individuals·m^−2^. Per plant species, LIDDU presented 465 ± 251 individuals·m^−2^ on average, while CARHI represented 560 ± 223 individuals·m^−2^. The per tray position mean values (LIDDU + CARHI) were 1128 ± 240 and 929 ± 256 individuals·m^−2^, for the border and inner trays, respectively. Noteworthily, the variability in density was higher in the inner than in the border trays. No significant differences were found between species nor tray position. 

### 2.2. The Growth Cycle of LIDDU 

Throughout the duration of the experiment, no plant species were observed in the control treatment, meaning that the substrate was free of plant propagules. Concerning the sown pots, the chronology of phenological events is shown in Table 2. Seeds germinated within a very short amount of time, and soon after, cotyledons appeared visible to the naked eye. Flowering started a little before a month had passed. As the plants developed leaf whorls, new flower buds arose in the leaf axils; thus, the stages of flowering and fruiting were almost contemporary to the stage of seed dehiscence, which was first noted on DAS 57 (DAS = Days After Sowing). According to these records, the length of the LIDDU reproductive cycle, i.e., from sowing to the start of seed dispersal, was nearly two months. Plants dried up from DAS 83 and were considered dead on DAS 100; that is, the duration of the growth cycle was estimated at 100 days in these experimental conditions. (See Supplementary Material S1).

Once the cycle was over, the whole plants (shoots + roots) were removed from the pots for growth measurements. Plant counts showed that stem density was equivalent to 6833 individuals·m^−2^ on average (7.1% coefficient of variation, cv), which would be equivalent to 14-times the population recorded for LIDDU in the nursery. The dry matter content of the above ground biomass was 26.5% (cv 1.4%); its dry weight was equivalent to 368.8 g·m^−2^ (12.7% cv). The ratio of the shoots to roots was 6.3 (dry weight basis). Mean growth rate—from sowing to senescence—was equivalent to 19.4 g·week^−1^·m^−2^.

### 2.3. Germination of LIDDU Seeds 

The start of the germination (germination time) was observed on Day 2 for the naturally-dehisced seeds in the 25 °C treatment, whereas in the 20 °C treatment the germination started on Day 3. The first observations of open cotyledons were made on Day 4 (19% of the germinated seeds) and Day 6 (1%), respectively. In the 25 °C treatment, the first hypocotyl fibrous root started to grow on Day 10, whereas this observation was recorded on Day 17 for the other treatment (see Supplementary Material S1). The germination period (number of days from the first observed germination to the last one) was 2.3 times higher in the 20 °C treatment than in the 25 °C one.

**Figure 3:**
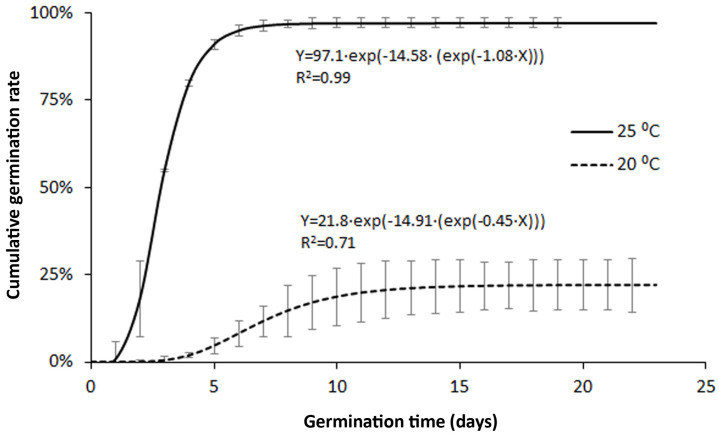
Fitted curves for the germination of naturally-dehisced seeds of *Lindernia dubia*. The bars show ±standard deviation values.

The time course of germination in the two temperature treatments is shown in Figure 3. Significant differences were observed between the results of both treatments, especially in final cumulative rates: 97.8 ± 1.4% at 25 °C versus 22.8 ± 7.7% at 20 °C. In addition, the variability within replicates was usually higher in the 20 °C treatment. Consistent with this, the fit of data to the Gompertz model gave better coefficient of correlation for the 25 °C treatment (r = 0.9948) than for the 20 °C one (r = 0.8435). 

With regard to the whole fruit seeds (WFS), the germination at 25 °C resulted in a 71.8 ± 6.6% final germination rate on average. The asymptote in the fitted germination curve took the value of 69.8%, while the coefficient of correlation was 0.9656.

### 2.4. LIDDU Traits

**Figure 4:**
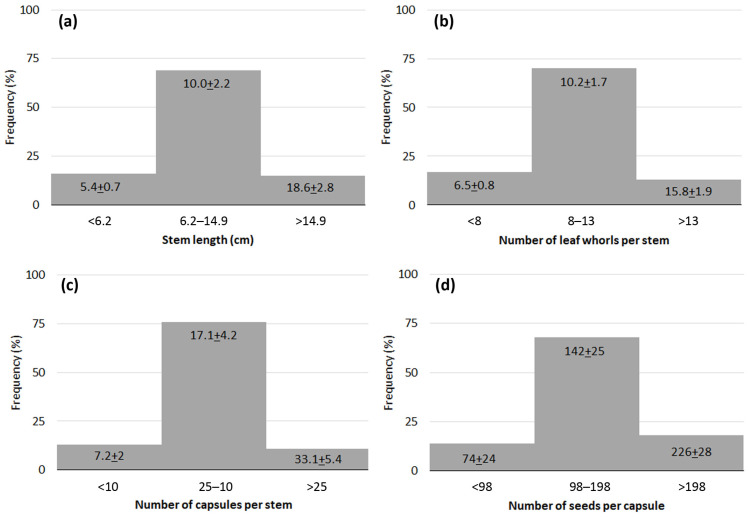
Frequency diagrams for data on traits of *Lindernia dubia*: (**a**) stem length; (**b**) number of leaf whorls per stem; (**c**) number of capsules per stem; (**d**) number of seeds per capsule.

**Figure 5:**
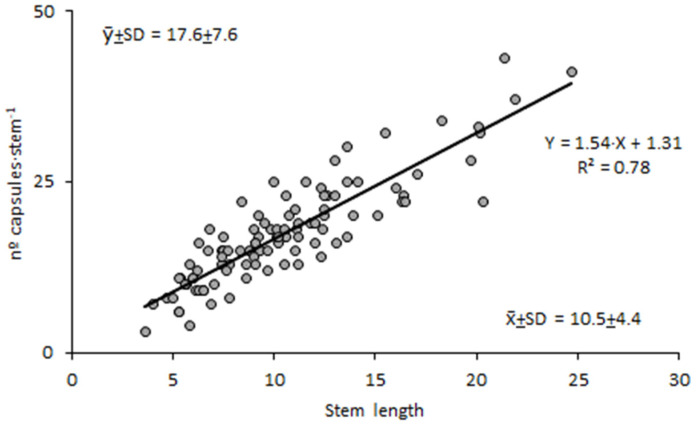
Relationship between the number of capsules and the length of the stem of *Lindernia dubia*.

The stem length of LIDDU plants that accompanied cattails ranged from 3.6 to 24.7 cm (10.5 cm on average, 41% coefficient of variation) (Figure 4a) and exhibited from five to twenty-one leaf whorls (Figure 4b), which meant about one leaf whorl per cm. The number of capsules per stem also varied greatly, from three to forty-three, in line with the variability found in stem length; on average, there were 17.6 capsules per stem (43% c.v.) (Figure 4c). Nevertheless, there was a close relationship between the number of fruits and stem length (Figure 5), suggesting a regular pattern of flowering and fruiting linked to leaf whorls. Seed production also varied greatly among capsules; it ranged from 34 to 292 seeds, with a mean value of 149.4 seeds·capsule^−1^ (30% c.v.) (Figure 4d).

**Figure 6:**
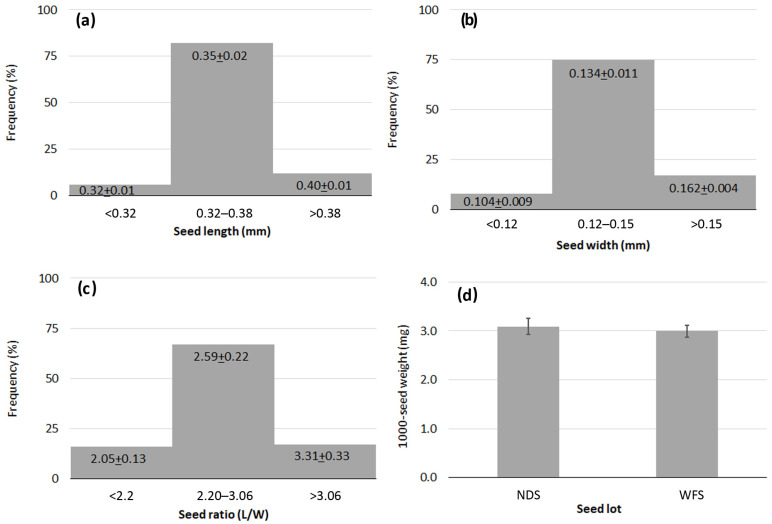
Frequency diagrams for data on traits of *Lindernia dubia*: (**a**) Seed length; (**b**) Seed width; (**c**) Seed ratio length to width; (**d**) 1000-seed weight. NDS = naturally-dehisced seeds; WFS = whole fruit seeds. The bars show ± standard deviation.

The variability found in the seed measurements was low, showing that the seeds were rather uniform in size and mass, and tiny. The mean seed length and seed width were 0.35 mm (9% c.v.) (Figure 6a) and 0.14 mm (13% c.v.) (Figure 6b), respectively. The ratio of seed length to width ranged from 1.60 to 4.22, with a mean value of 2.6; this value reflected the rectangular seed shape (Figure 6c). The weight of a thousand seeds varied from 2.86 to 3.27 mg; the NDS lot was slightly heavier (3.09 ± 0.17 mg) than the WHS lot (3.00 ± 0.12 mg), but the difference was not statistically significant. The mean value for 1000-seed weight was 3.04 mg (Figure 6d).

The potential seed production of a plant, or plant seed number, was estimated at 2629 seeds·plant^−1^ from the mean values found for the number of capsules per stem and the number of seeds per capsule; this amount would mean that the population seed number in the seedling trays might achieve over 1.2 × 10^6^ seeds·m^−2^ at the end of the growth cycle. In order to assess the reproductive capacity of a plant in the absence of competence, a 97.1% optimum germination rate (value of the asymptote on the germination fitted curve of NDS at 25 °C) and a 72.7% seed dispersal rate (from the asymptote values of NDS and WFS germination fitted curves) were assumed. With these assumptions, the maximum reproductive capacity (sexual propagation) was estimated at 1857 individuals in a cycle of about three months, as long as temperature, substrate and water were not limiting factors.

## 3. Discussion

Recently, the spread of alien species has become a matter of concern, especially when they present invasive traits. In an agricultural or horticultural environment, the record of an alien plant species may represent a potential weed infestation and/or a risk for nearby ecosystems. One of the main goals of this work was to gain insight into the spontaneous flora occurring in cattail nurseries. Certainly, the use of potted cattail seedlings infested with weeds found in constructed wetlands may result in the spread of undesirable plant species, including potential invaders. 

In the present work, the inventory of species accompanying cattail seedlings—grown in a substrate free of plant propagules—revealed a low amount of diversity; it also showed that such species have a preference for moist habitats [22]. Thus, CYPER was reported as the most frequent alien species on the river banks of the Cantabric watershed in Spain [23]. AGSST is widely distributed in Europe and Spain, where it appears in humid meadows [22]. CYPRO and ECHCG have been acknowledged as major weeds of rice fields globally [11], and DIGSA has been reported as a weed in rice fields of India and USA [11]. In Spain, CYPRO, ECHCG and SONOL were reported as the main weeds in sprinkler-irrigated rice [24]. In a study of the success of alien weeds in irrigated Mediterranean orchards, CYPRO and ECHCG were categorized as ‘high successful’ invaders [25]. Notwithstanding the above, the constancy values of the above-mentioned species were low in our case study; the dominant species were others such as LIDDU (first in the rank) and CARHI.

To the best of our knowledge, the occurrence of the alien species LIDDU in our area of study (Madrid, Spain) has not been reported so far [21,26,27]. According to the Information System of the Plants of Spain [21] the closest record of the occurrence of LIDDU was located in the proximities of the Rosarito reservoir, i.e., 138 km from our location. It is worth noting that this species was cited as a global plant invader by Laginhas and Bradley [28], and that it was reported as an invasive plant species in some regions of Europe [5]. In eastern Spain (provinces of Tarragona and Valencia) it was reported within the list of emergent rice field flora (flooded rice crops), although with little presence [29]. The results for seed weight in this work and the literature data [30,31] showed that LIDDU seeds are lightweight so, the accidental introduction of this alien species into our region might have occurred through a wide range of pathways. It could have even occurred due to waterfowl, a pathway suggested by Lovas-Kiss et al. [32] for LIDDU dispersal in Hungary, and by Soons et al. [33] for small seeds in general. In this respect, it is worth noting that our facilities are about 500 m from the Manzanares river (closest river).

CARHI (hairy bittercress) is a well-known weed species, which is widely studied in the literature. Vaughn et al. [34] stated that CARHI was a major pest in nurseries in USA and highlighted that CARHI occurred “in patches in nursery crops and can solidly fill nursery pots if left uncontrolled” (sic). Warnings about CARHI as a weed of plant nurseries have also been launched in Spain at least since 1997 [35]. Vaughn et al. [34] highlighted large seed production (up to 5000 seeds per plant), quick generation times (30–60 days per generation, in favorable climates) and enhanced mechanisms of seed dispersal (ballistic seed dispersal) as competitive attributes of CARHI. 

The occurrence of LIDDU and CARHI across seedling trays seemed to be influenced by the proximity to the corridors, although no statistically significant differences were found due to the high variability in the counts, e.g., LIDDU presented a 63% coefficient of variation for plant counts in the inner trays. Despite the variability, it was noticed that LIDDU performed a little worse in cells all around surrounded by cattails (i.e., in the inner trays). This observation is considered to be in line with the results of Simon and Jansen’s [5] work, who inferred that LIDDU would not easily out-compete fast-growing erect plant species; however, these authors suggested that the impact of LIDDU on small or minute plant communities could be adverse. Competition could explain the differences observed between tray positions in the present study. Another explanation could be the unintentional human contribution to seed dispersal (workers walked through the corridors) and wind dispersal; open corridors may result in preferential air flow paths, entailing a higher amount of seed dispersal towards the closest trays.

In vitro germination tests showed that the seeds of LIDDU have the potential to germinate very quickly at 25 °C, and that the germination rate is good, especially for naturally dehisced seeds. Interestingly, the growth of hypocotyl roots was observed at an early plantlet stage (see Supplementary Material S1), an observation that has not been previously reported in the literature. The only datum on LIDDU germination found in published scientific works was the 63% germination rate reported for the control treatment in the study of waterfowl seed dispersal by Lovas-Kiss et al. [32]; their test was conducted at room temperature (20–25 °C) and with a 16 h light/8 h dark photoperiod. Consistent with those temperature conditions, the value reported was between the rates we found at 25 °C and 20 °C in this work. Furthermore, the trends observed in our work seem to be consistent with previous studies of freshwater wetlands and other aquatic habitats that highlighted the capacity of LIDDU to germinate over other species present in seed banks [14,36]. In our tests, the germination rate sharply decreased at 20 °C, a fact that pointed to thermophilic preferences of this species. Although our findings refer to seed lots from a single location, the response of seed germination to temperature obtained in this work suggests that infestation by LIDDU is not likely to happen in the cold season in temperate climates, at least in the short period of time since the introduction of this species. Nevertheless, Simons and Jansen [5] categorized LIDDU as a naturalized invasive in the Netherlands (temperate Atlantic north-western European climate), but estimated that the transition from being a casual alien plant to that status could have taken about ten years. Additionally, there is evidence of the environmental impact of LIDDU in warmer countries within Europe such as Italy and Romania [3]. 

The chronology of phenological events and plant traits data presented in this work suggest that LIDDU possesses competitive attributes such as morphological variability, early flowering, long seeding time, short growth cycle, small and light seeds, and high seed production. Peralta and Royuela [37] cited that LIDDU had a summer phenology in North Spain, flowering from July to September, like we observed in central Spain. The botanical description accompanying the data for LIDDU in North America from [38] reads “flowering year-round”; Baker [31] observed that LIDDU flowering ended in autumn (September) in California. With regard to plant size, the values in this work were in line with the botanical description of LIDDU by Lewis [1]. The results for seed length and width were consistent with the taxonomical study of Linderniaceae conducted in Korea by Bazarragchaa et al. [39], and also with data provided in the study of endozoochorous seed dispersal in Hungary [32]. Bazarragchaa et al. [39] described the inflorescence of LIDDU as occurring in axillary flower pairs, solitary axillary or branching in the subtending leaf. Despite the fact that the number of capsules in a single stem was not reported by those authors, it can be inferred that the finding of nearly two fruits (capsules) per leaf whorl in our work is in line with their inflorescence description. Data on capsule seed number or plant seed number were not found in the consulted literature. Some data on 1000-seed weight were found but appeared controversial to a certain extent. Thus, the Seed Information Database [30] reports 0.01 g as the mean value for 1000-seed weight, whereas the Baker Seed Collection database, 0.003 g for the same number of seeds [31]. However, a note on the former value says that minor covering structures could have been included in the weights. Therefore, our results for 1000-seed weight are consistent with Baker’s, and support the hypothesis that minor covering structures were included in the weights reported for LIDDU by the Seed Information Database [30]. 

In the literature, there has been much discussion on what plant traits can be associated with invasiveness, as well as on the dependence of plant traits on the environmental context; however, it is widely accepted that traits related to fecundity and species dispersal are essential to assess the potential success of an invader [40]. Concerning weed fecundity, Norris [41] underlined the risks of extrapolating data from controlled conditions to field conditions, but at the same time acknowledged that many papers report experiments carried out under artificial conditions; in addition, this author encouraged the collection of data for more weed species. In this sense, our estimates of plant seed production and maximum plant reproductive capacity should be taken with caution; however, they provide a basis along with the other results from this work for the estimation of the weedy potential of LIDDU.

## 4. Materials and Methods

### 4.1. Site Description

This study was developed at the nursery of aquatic plants of the Technical University of Madrid (UPM, Spain) (40°26′36″ N, 3°44′18″ W, 650 m a.s.l.). The climate at the site is temperate with dry and hot summers; normal values of temperature are 14.6 °C annual mean temperature, −7.4 °C absolute minimum temperature (January) and 40 °C absolute maximum temperature (August) [42]. 

The aquatic plant facilities included experimental wetlands, a greenhouse for plant propagation and laboratories for plant characterization. These facilities were intended to produce cattail plants for the establishment of externally constructed wetlands, as well as for research on phytodepuration. The greenhouse was endowed with an automated system for ventilation (20 °C set point); in addition, doors remained open during summertime. In the interior of the greenhouse, benches were conventionally arranged: two adjacent rows of benches in the middle (central rows) plus two separate rows at each side of the central rows with two aisle spaces of 1.10 m width in between. Benches were watertight by means of a polyethylene (PE) pond liner. They were periodically filled up with tap water to simulate shadow ponds. Every year in the period from 2017 to 2023, cattails were raised from seed in 96-cell plastic trays (52.5 × 33 × 8 cm) placed in the simulated water ponds; before seeding, trays were filled with a commercial substrate (Pindstrup Mosebrug S.A.E. code 51212, Burgos, Spain). After emergence, seedlings were thinned to one per cell and let to grow until planting in constructed wetlands. The alien LIDDU was noticed for the first time in the summer of 2022. 

### 4.2. Weed Inventory and Assessment of the Dominant Species

The occurrence of spontaneous plant species in cattail seedlings was studied in autumn 2022. At that time, cattails were about 50–65 cm tall and exhibited 5–7 green leaves. Identification of spontaneous plant species growing in the trays was carried out using botanical keys and monographs [22,43]. Species constancy was assessed as the ratio of the number of seedling trays with a determined species to the total number of seedling trays (*n* = 174). The indexes of Shannon (H) [44] and Simpson (D) [45] were calculated from tray counts in order to estimate species diversity and dominance.

The occurrence of LIDDU was assessed in terms of population density and distribution across the nursery, as compared to CARHI, the other dominant weed species in this study. A randomized block design with four blocks and two replications per block was used. To this end, two categories, border (trays placed next to the aisle) and inner (trays placed on the inner area of the bench table), were made. Population density (individuals·m^−2^) was calculated from the number of individuals counted in each seedling tray, considering the tray surface area (1536 cm^2^·tray^−1^).

### 4.3. Growth Chamber Experiments

Two types of experiments were developed in a plant growth chamber (300 L capacity, forced air circulation, 6 × F30W/Gro-Lux T8) to gain insight into LIDDU growth cycle and seed germination. 

The first one was a pot experiment, where the pot dimensions were top diameter, 10.5 cm; bottom diameter, 8.5 cm; and height, 9.5 cm. Pots (*n* = 4) were filled up with the same substrate as the one used in the cattail nursery (Pindstrup Mosebrug S.A.E. code 51212). A control (no sown treatment) was used to check the absence of plant propagules in the substrate. Mature fruits (light-brown capsules, starting to break) were taken from senescent LIDDU plants in the seedling trays; seeds were spread on the pot surface, using 50 capsules·pot^−1^; capsules were left on the ground. Then, the just-sown pots were placed in a water bath inside the growth chamber at 25 °C constant temperature and in a 14 h day/10 h dark cycle. Air humidity was maintained ≥79.1% throughout the experiment. Water level in the bath was kept at pot surface level (daily water replenishment). Dates of the start of the phenological stages visible cotyledons, flowering, fruiting, seed dehiscence and plant senescence (Supplementary Material S1) were recorded and expressed as DAS (Days After Sowing). Once the seed dehiscence was observed, naturally dehisced seeds from the potted plants were collected on a daily basis; after seed cleaning (debris separation under stereomicroscope) they were gathered in a seed lot named NDS (=naturally dehisced seeds). When plants were fully dry (brown-colored shoots), they were uprooted in order to determine the following parameters: population density (individuals·m^−2^), shoots dry matter content (%), average growth rate (dry mass accumulated per week and ground area, g·week^−1^·m^−2^) and the ratio of shoots/roots (dry weight basis). Dry weights were determined after oven-drying at 105 °C. 

The other type of experiments was in vitro seed germination tests. Germination of NDS was performed in covered Petri-dishes filled with filter paper moistened with distilled water; 2 thermal regimes (20 and 25 °C constant temperature) were tested using three replicates of about 100 seeds each (*n* = 3 × 100). The photoperiodic regime was 14 h light/10 h dark. Observations were made on a daily basis under a Leica EZ4 stereomicroscope (Wetzlar, Germany) 35 × (transmitted illumination). A seed was considered to have germinated when the radicle emerged (see Supplementary Material S1). The germination rate was calculated as the number of germinated seeds divided by the total number of seeds in the Petri dish. Dates of the first observation of visible cotyledons and lateral root growth were annotated as well.

Another seed lot was prepared and tested for in vitro germination. All seeds (naturally dehisced plus not-dehisced seeds) inside 50 mature LIDDU capsules were extracted by shaking and by hand with the help of a thin brush and a spatula; after seed cleaning under stereomicroscope, seeds were gathered in a seed lot named WFS (Whole Fruit Seeds). WFS germination was tested at 25 °C constant thermal regime, following the same procedure as for NDS tests. Final germination rate of WFS was compared to that of NDS in order to assess the seed dispersal rate of LIDDU capsules. 

### 4.4. LIDDU Traits

Plant traits studied were the following: stem length (SL), number of whorls per stem (NW), number of capsules per stem (NC), seed production per capsule (NS), seed size, 1000-seed weight, and maximum reproductive capacity in absence of competence. One hundred shoots in the senescence stage (brown shoots) were randomly sampled from cattail seedling trays in the nursery for SL, NW and NC; from these plants, fifty capsules—dry capsules starting to open—were taken at random for NS. Seed extraction and seed cleaning (separation from debris) were performed by hand with the help of laboratory equipment (thin brushes, tweezers, spatulas, stereomicroscope); afterwards, seeds from each capsule were counted under a stereomicroscope to determine NS. Seed size measurements included seed length (L) and width (W) and the ratio of L/W [39]. The sample size for seed measurements was one hundred seeds. Thousand-seed weight (air-dry seeds) was determined in triplicate using a five decimal precision balance. This trait was studied for the two lots of seeds, NDS and WFS (*n* = 3 × 2 × 1000). 

The maximum reproductive capacity (individuals per ground area, pl·m^−2^) was calculated as the product of mean NS, mean NC, seed dispersal rate and optimum germination rate. It was assumed that the optimum germination rate was the highest asymptote value found for the germination curves in the germination tests.

### 4.5. Statistical Analysis

Descriptive statistics (mean, standard deviation, minimum, maximum, coefficients of variation) were calculated. Data for LIDDU traits were graphically presented in terms of frequency (percentage in a category). Population densities of dominant species were analyzed by one-way ANOVA with the location of plants in the nursery as between-subject factor and two weed dominant species (factor with two levels) as a within-subject factor. For the study of seed germination, data were fitted to the S-shaped Gompertz function: Y = a × exp(−b × exp(−c × T), using the cumulative germination rate as dependent variable and the germination time (days) as independent variable; parameters a, b and c in the Gompertz function were calculated by successive iterations. Data analyses were conducted using the statistics software Statgraphics 19^®^.

## 5. Conclusions

The occurrence of the alien plant species *Lindernia dubia* (L.) Pennel, as a dominant weed in cattail seedlings, has been reported in Madrid (Central Spain) for the first time; its introduction might have occurred due to waterfowl and wind. The population density attained by *L. dubia* has been found to be comparable to that of *Cardamine hirsuta* L., a well-known weed species in plant nurseries. The characterization of *L. dubia* has provided evidence that this species presents the following competitive attributes: variability in stem length, number of capsules per plant and number of seeds per capsule; short growth cycle (assessed at 25 °C); ability to flower at an early stage of growth; long seeding time; small and light seeds; high seed production; and a high germination rate at 25 °C; all of this suggests a high reproductive capacity in a short period of time. On the whole, the dataset obtained in this study has provided a basis for understanding the weedy potential of *L. dubia*, and for management decisions of this potentially invasive species in Europe.

## Figures and Tables

**Table 1:** Inventory of plant species present in cattail seedling trays and constancy values.

Taxon	EPPO Code ^1^	Family	Life Cycle	Origin	Constancy
*Lindernia dubia* (L.) Pennel	LIDDU	Linderniaceae	Annual	America	0.9828
*Cardamine hirsuta* L.	CARHI	Brassicaceae	Winter annual	Europe	0.9425
*Cyperus eragrostis* Lam.	CYPER	Cyperaceae	Perennial	Tropical America	0.3391
*Epilobium roseum* Schreb.	EPIRO	Onagraceae	Perennial	Europe to China	0.2471
*Agrostis stolonifera* L.	AGSST	Poaceae	Perennial	Eurasia, North Africa	0.0862
*Digitaria sanguinalis* (L.) Scop	DIGSA	Poaceae	Summer annual	Mediterranean region to Central Asia and Malaysia	0.0632
*Cyperus rotundus* L.	CYPRO	Cyperaceae	Perennial	Indian sub-continent	0.0287
*Sonchus oleraceus* L.	SONOL	Asteraceae	Summer annual (biennial)	Mediterranean region, Europe	0.0230
*Echinochloa crus-galli* (L.) P.Beauv.	ECHCG	Poaceae	Summer annual	Europe	0.0172

^1^ EPPO = European and Mediterranean Plant Protection Organization https://gd.eppo.int (accessed on 20 September 2023).

**Table 2:** Chronology of phenological observations. *Lindernia dubia* grown from seeds at 25 °C. DAS = Days After Sowing.

DAS	Start of Phenological Stage
6	Visible cotyledons
15	Leaf development
30	Flowering
42	Fruit development
57	Seed dehiscence
83	Senescence
100	Dead plants

## Data Availability

Data are contained within the article.

## Supplementary Materials

The following supporting information can be downloaded at: https://www.mdpi.com/article/10.3390/plants13131859/s1, Supplementary Material S1: Photographic report.
