# Supplementary material for: Lindernia dubia (L.) Pennel as an Alien Weed in Central Spain: A Case Study
Source: Plants (Basel). 2024 Jul 5;13(13):1859. doi: 10.3390/plants13131859 (PMC11244003; doi:10.3390/plants13131859)
Supplement: Supplementary file 1 [file plants-13-01859-s001.zip › Supplementary material S1 CURT.pdf]

Supplementary material S1

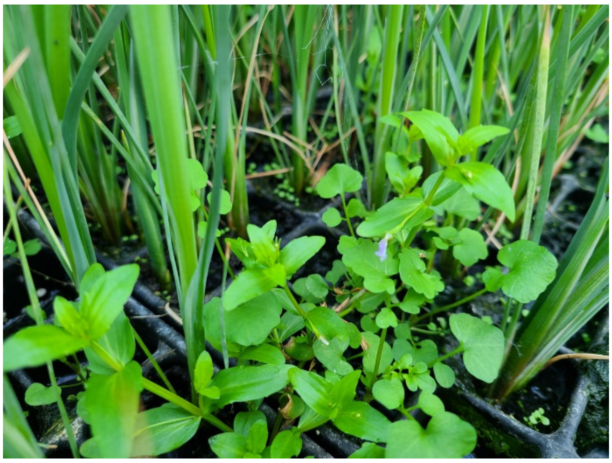	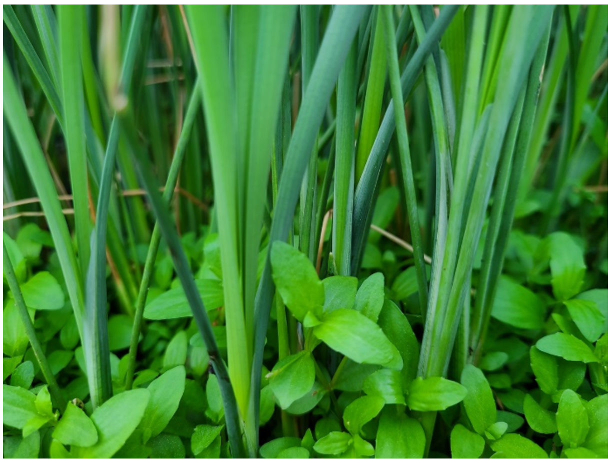
The occurrence of LIDDU & CARHI in seedling cells. Nursery experiment.	Detail of LIDDU in seedling cells. Nursery experiment.
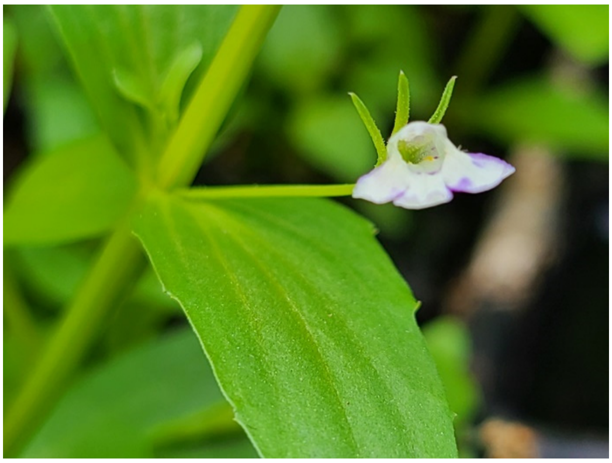	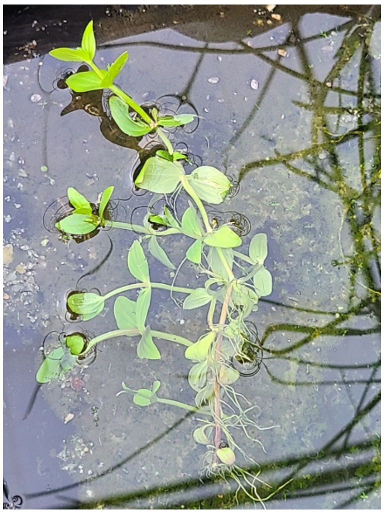
Detail of a flower of LIDDU. Nursery experiment.	LIDDU stem rooting at the lower nodes. Nursery experiment.
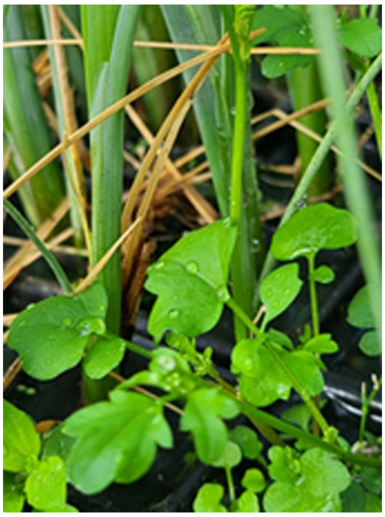	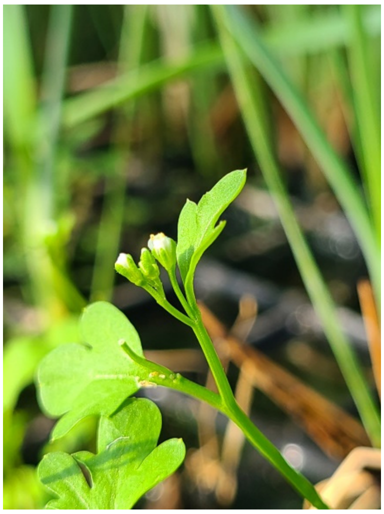
Detail of CARHI in seedling cells. Nursery experiment.	CARHI flowering and fruiting. Nursery experiment.

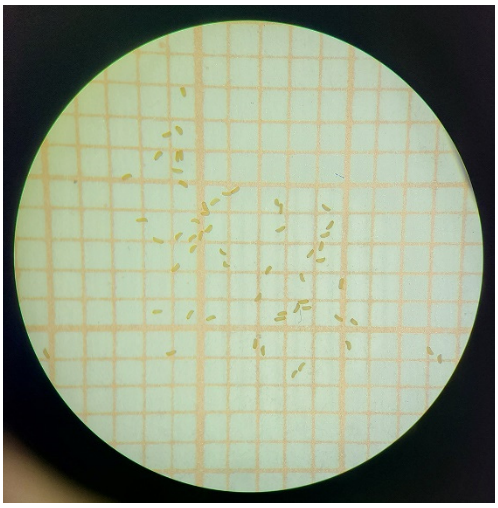	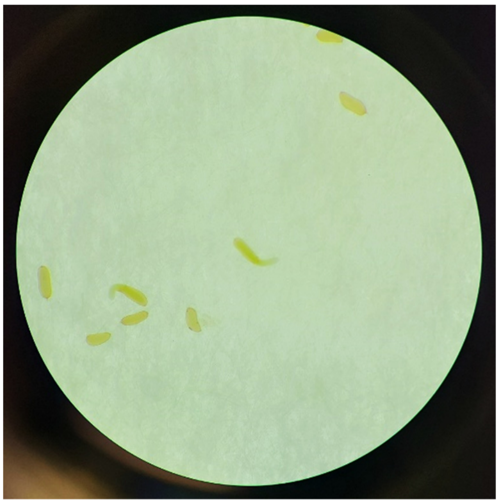
LIDDU seeds. Seed length 0.3-0.4 mm.<br>In-vitro germination tests.	Emergence of radicle. LIDDU seeds.<br>In-vitro germination tests.
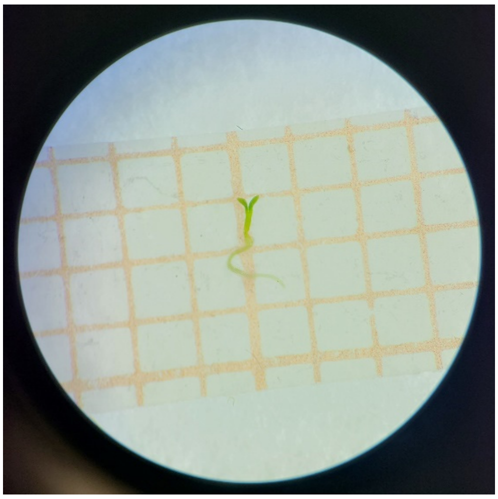	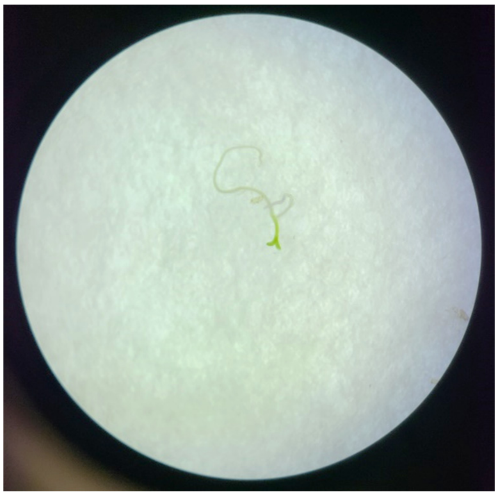
Open cotyledons. LIDDU plantlet.<br>In-vitro germination tests.	Hypocotyl fibrous root growth. LIDDU plantlet.<br>In vitro germination tests.
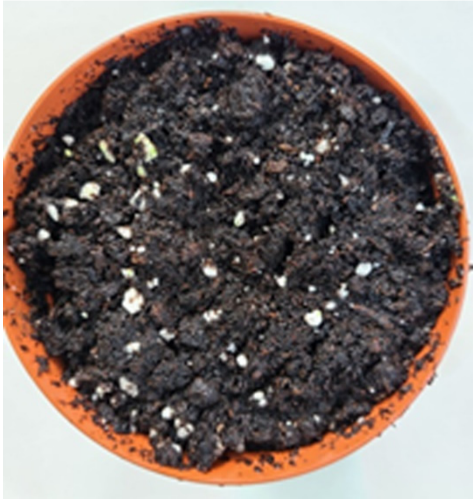	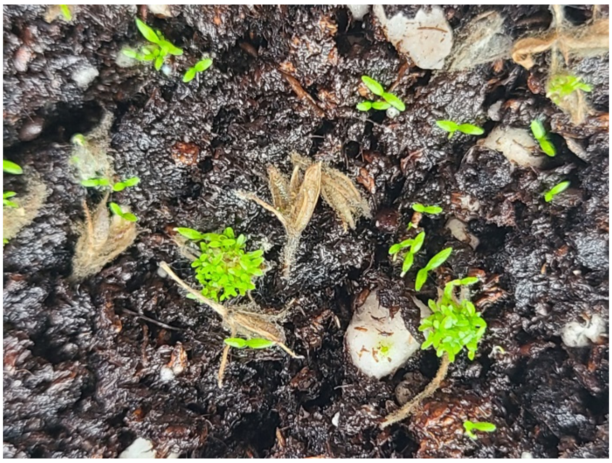
Pot experiment. Control treatment.	Emergence of the first plantlets of LIDDU.<br>Open cotyledons. Pot experiment.

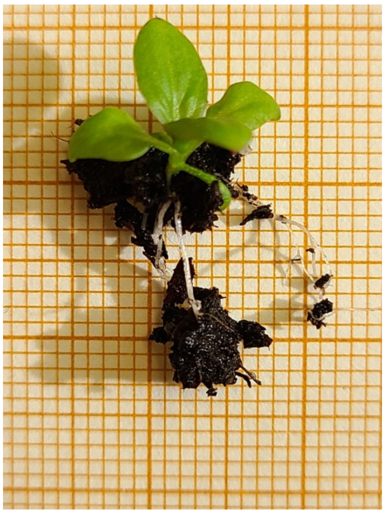

A plantlet of LIDDU at the stage of first true leaves. Growth of fibrous root system.
Pot experiment.

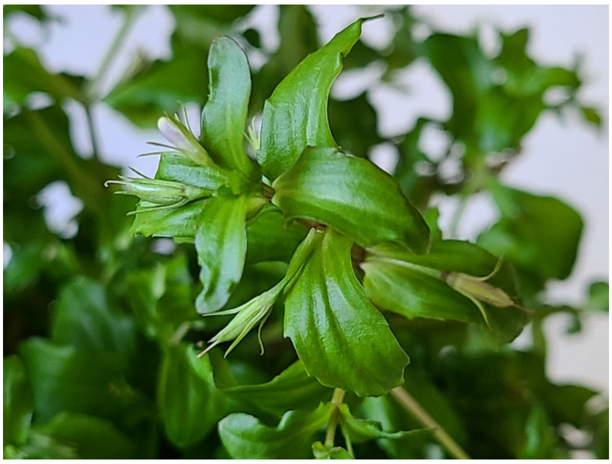

Co-existence of flowers and fruits in LIDDU.
Pot experiment.

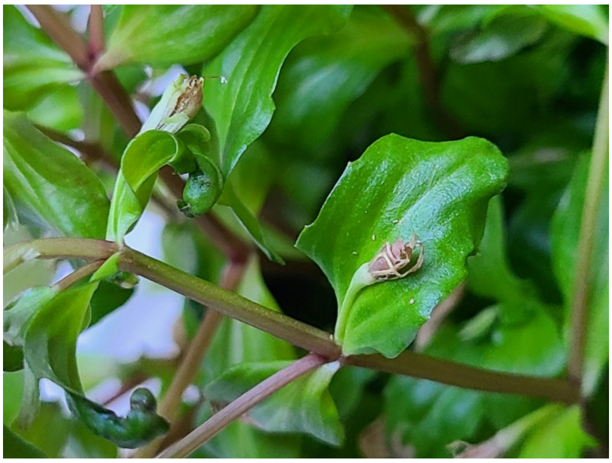

Mature capsules of LIDDU and seed dehiscence. Pot experiment.

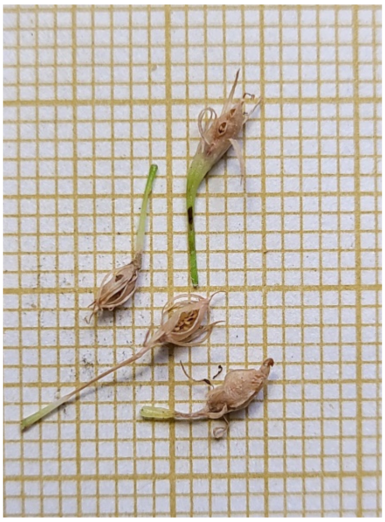

Capsule opening.
Pot experiment.

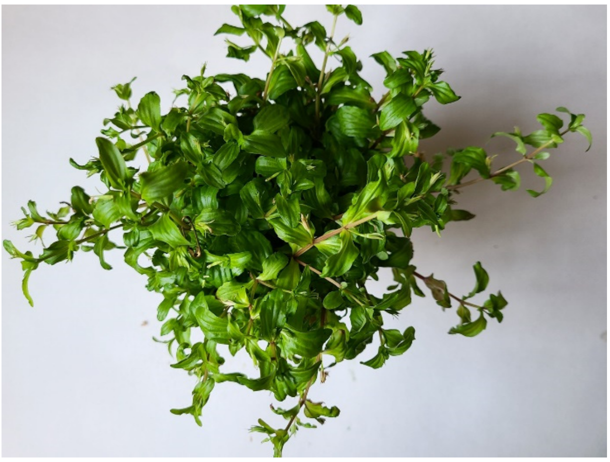

Live plants of LIDDU.
Pot experiment

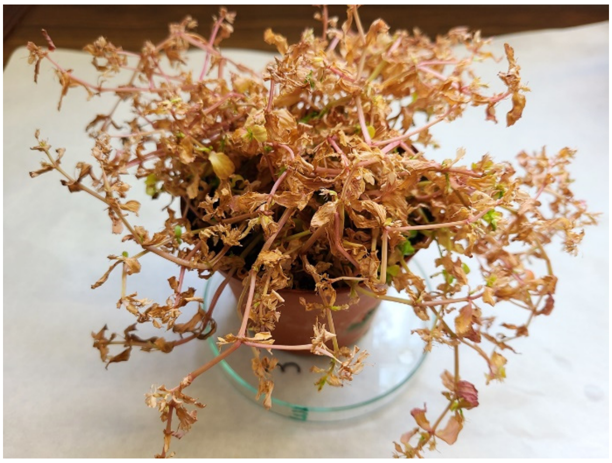

End of LIDDU plant cycle.
Pot experiment.
